# Thyroid Function and Mood Disorders: From Etiology to Treatment Response

**DOI:** 10.1192/j.eurpsy.2026.10602

**Published:** 2026-07-31

**Authors:** D. Kolar, M. Cho, M. V. Kolar

**Affiliations:** 1 Department of Psychiatry, Queen’s University, Kingston, Canada; 2 Medicine, Princess Alexandra Hospital, Brisbane, Australia; 3 Internal Medicine, Wayne State University School of Medicine, Detroit/Rochester Hills, United States

## Abstract

**Introduction:**

Thyroid hormones regulate mood, brain development and neurotransmitter systems. The association between thyroid dysfunction and major mood disorders, including major depressive disorder (MDD) and bipolar disorder (BD) has been extensively studied, but it is unknown whether these associations are causal or not. Thyroid dysfunction, both hypothyroidism and hyperthyroidism, is strongly linked to developing mood disorders. Thyroid hormones show potential as biomarkers in mood disorders by predicting treatment outcomes.

**Objectives:**

We performed a literature review on the association between thyroid hormones, thyroid dysfunction and developing mood disorders, as well as on the role of thyroid hormones as a biomarker of treatment response in mood disorders.

**Methods:**

We searched the electronic databases PubMed, PsycINFO and EMBASE to identify appropriate studies. All relevant articles over the last 20 years were included. Search terms included thyroid hormones, thyroid dysfunction, mood disorders, depression, bipolar disorder, and biomarkers.

**Results:**

Many studies have shown that thyroid dysfunction is associated with depression. Hypothyroidism has been considered a strong risk factor for developing depression. However, newer studies have shown that the association between hypothyroidism and clinical depression is considerably lower than previously assumed. Several studies have proposed that thyroid hormones may have a role in the etiology of bipolar disorder. Increased prevalence of thyroid autoantibodies in bipolar patients indicates that a thyroid autoimmunity present in 20-30% of patients with BD might be a genetic marker of vulnerability for bipolar disorder. It is also important to recognize the thyroid hormone mechanisms beyond the HPT axis and the role of deiodination, the conversion of T4 to biologically active T3, and the genetic variation of thyroid deiodinase enzymes in patients with depression. Injection of T3 increases neurogenesis by upregulating BDNF. With respect to thyroid function and treatment response in mood disorders, in some cross-sectional studies of patients with MDD, higher FT4 concentrations were associated with more severe depression, while a significant decrease in T4 was associated with treatment and clinical improvement. T3 augmentation in MDD with partial response to antidepressants is usually effective with a 50% improvement rate. T4 augmentation is effective in patients with bipolar disorder. Higher doses of T4 are helpful in rapid cycling bipolar disorder. Finally, brain-targeted thyromimetic ABX-002, as a CNS-directed thyroid hormone receptor beta agonist, is currently studied as an adjunctive treatment for MDD and BD.

**Conclusions:**

Although there is no definite answer about the causal relationship between thyroid dysfunction and mood disorders, the role of thyroid hormones in mood regulation and response to antidepressant treatments is significant.

**Disclosure of Interest:**

None Declared

